# Cases

**Published:** 1881-02

**Authors:** E. W. Berridge

**Affiliations:** London


### SOME CLINICAL CASES.

BY E. W. BERRIDGE, M.D., LONDON.

1. Sulphur.—Nov. 20th, 1880. A boy, aged seven and a half, was brought to me at 9.30 A.M., with the following symptoms, which had appeared the same morning: End of prepuce considerably swollen, red, and partly retracted, exposing glans. "Allen's Index to the Encyclopedia" had recently arrived; this was a good opportunity for testing its utility. On referring to page 891, I found that only *Sulphur* corresponded to all three symptoms, *i. e.*, redness, swelling, retraction; while it also has smarting in morning after waking. The similarity being so complete, I gave only a single dose of *Sulphur* M.M. (F.C.) When I examined patient again, at 1.15 P. M., all the symptoms had disappeared, and he had been playing.

2. Carbo an.—May 6th, 1880. Two days ago Mrs. ——, while playing in the evening, struck her coccyx violently against the sharp edge of a piece of furniture, having at the time only her night-dress on. The pain made her feel sick at her stomach and faint. *Arnica* was applied locally, but the pain steadily increased. I saw her next day, at 10.30 A. M.; there was an aching and burning pain in coccyx, which was also very tender to the touch; pain in coccyx, on stepping with right leg; it was better when standing with right foot raised from the ground; last night could not lie on right side on account of the pain. In C. Lippe's invaluable repertory, I found (p. 204), "Pain in os coccygis when touched, *carbo an.*, *Kali bichr.*, *Lach.*" Of these only *carbo an.* has burning in coccyx, therefore I selected it as the *simillimum*. As, however, the conditions of the case had not been observed under this remedy, I was not sure that a single dose would prove sufficient. I, therefore, dissolved some globules of *carbo an.* 3 M. (Jenichen) in water and ordered a spoonful, to be taken, every three hours till better. Saw her again at 10 P. M.; she felt relieved soon after the third dose. When I saw her the aching was very much better, but the burning and tenderness yet remained. She could sit with much more comfort and could rise more easily from a seat. Could not use right leg yet, and the pain returned on stretching right arm out, as well as from treading on right heel. Faintness and nausea were much less. I did not consider the improvement sufficiently marked to stop the remedy, therefore it was continued every four hours, till decidedly relieved.

May 7th, 9 A. M. Has taken two doses, one last night and one this morning. Is much better, can move without pain. Stopped medicine.

May 8th. The burning and aching all gone, parts still tender, can stand on right leg without pain.

May 9th. More tender to-day, having stood much yesterday; can put foot to ground naturally.

May 10th. Much better; walked out doors, and soon recovered. In this case the routine practice of applying *Arnica* to a bruise, failed; the symptoms were cured by the internal administration of the dynamized homœopathic remedy.

3. Lachesis.—Mrs. —— had after pains, beginning in left lumbar region, going around left side, across to right side of abdomen, and down left thigh. Before I arrived the nurse, whom I had instructed in true Homœopathy, gave a dose of *Lachesis*, very high. On examining the symptoms, the remedy seemed well indicated by the direction of the pain from left to right; though *Lac Caninum* has ("C. Lippe's Repertory," p. 155) "after pains going down to thighs." I ordered the *Lachesis* to be repeated as occasion might demand, leaving *Lac Can.* in case it failed. The *Lachesis* cured without the need of any other remedy.

4. Lac Vaccinum Defloratum.—While nursing the patient just mentioned, the nurse was attacked with the following symptoms: boring pain, sore as if bruised and throbbing in right kidney, extending as a dull aching to right hip, to spine, up to right shoulder, and across right side of abdomen to shoulder; afterward the boring affected the left kidney also, with a little aching there; backache across sacral region, much worse in the centre—a breaking pain; all the pains worse from movement; afterward sensation as if abdomen were full of water, catching the breath like a spasm. After suffering for twenty-four, she took *Lac Vacciuhm Defloratum* 200; in three hours she was better, and was speedily cured.

Some months ago she cured herself of a similar attack with the same remedy. The selection was made according to Dr. Laura Morgan's clinical case reported in *The Organon*, vol. ii, pp. 255–6.

