# Errata

**Published:** 1881-04

**Authors:** 


### ERRATA.

In Dr. Berridge's clinical article (Feb. number) there are a few errors; p. 68, line 2, for *next*, read, *on*; p. 69, line 14, for first *shoulder* read *scapula*; for second *shoulder* read *bladder*; p. 69, line 14 after *24* add *hours*.

